# Improvement of the Shelf-Life Status of Modified Atmosphere Packaged Camel Meat Using Nisin and *Olea europaea* Subsp. *laperrinei* Leaf Extract

**DOI:** 10.3390/foods9091336

**Published:** 2020-09-22

**Authors:** Djamel Djenane, Malek Aboudaou, Fatiha Djenane, Diego García-Gonzalo, Rafael Pagán

**Affiliations:** 1 Laboratory of Food Quality and Food Safety, Department of Food Science, Mouloud MAMMERI University, P.O. Box. 17, Tizi-Ouzou RP 15000, Algeria; d.djenane@hotmail.com; 2 Département Recherche & Développement, Isser Délice SARL, ISO 9 International, BP 10, 35230 Isser, Algeria; mapgdz@yahoo.fr; 3 Departamento de Producción Animal y Ciencia de los Alimentos, Facultad de Veterinaria, Instituto Agroalimentario de Aragón-IA2 (Universidad de Zaragoza-CITA), 50013 Zaragoza, Spain; Diego.Garcia@unizar.es (D.G.-G.); pagan@unizar.es (R.P.)

**Keywords:** one-humped camel, meat, *Olea europaea* subsp. *laperrinei*, Tassili n’Ajjer, nisin, O_2_/CO_2_, packaging, combined treatment, shelf-life

## Abstract

The impact of combined biopreservation treatment with *Olea europaea* subsp. *laperrinei* leave extracts (*laper*.OLE) and nisin on the quality attributes of camel steaks packaged under high O_2_ (80%) and CO_2_ (20%) atmosphere was investigated during refrigerated (1 ± 1 °C) long-term storage. As measured by reversed phase HPLC/DAD analysis, oleuropein is the phenolic compound most present in the chemical composition of *laper*.OLE (63.03%). Camel steaks treated with *laper.*OLE had a lower concentration of thiobarbituric acid-reactive substances (TBA-RSs) in the course of 30 days of storage. Surface metmyoglobin (MetMb) increased at a reduced rate in *laper*.OLE-treated samples compared to control samples. Neither modified atmosphere packaging (MAP) nor biopreservation treatments significantly altered the tenderness of camel steaks, expressed in terms of Warner-Bratzler shear force (WBSF), as compared to control samples. After 30 days of storage, psychrotrophic bacteria and *Pseudomonas* spp. counts were significantly lower in camel steaks treated with a combination of *laper*.OLE and nisin than in untreated steaks. Moreover, samples treated with *laper*.OLE received higher scores on bitterness acceptability. In sum, the use of combined biopreservation methods could be a sustainable solution for the preservation and promotion of the quality characteristics of camel meat in arid regions.

## 1. Introduction

In Algeria, the consumption of dromedary meat (one-humped camel) has increased significantly in recent years. For the Algerian government, the development of the camel meat sector has always been a priority in order to meet the needs of the population of the far south in terms of animal protein.

No up-to-date statistics on the number of tons of camel meat consumed in the region of Tassili n’Ajjer or the total in Algeria are available. However, and according to Food and Agriculture Organization (FAO) statistics, during the decade 2007–2017, the Algerian camel herd increased from 286,670 heads in 2007, to 315,000 heads in 2011, to 381,882 heads in 2017 [1]. This increase is the result of several camel breeding development programs implemented by the Algerian Government. With 5190 tons of camel meat produced in 2011, Algeria ranks fifteenth in the world for camel meat production, estimated worldwide at 356,000 tons. However, uncontrolled slaughter practices that escape the controls of the veterinary services are still very common. 

Camel meat is characteristically lean, with a lower fat content compared to other meat-producing animals such as bovines and sheep [2]. It also contains less cholesterol, along with a relatively high proportion of polyunsaturated fatty acids (PUFAs) [3]. This is an important factor in reducing the risk of cardiovascular disease, which is related to saturated fat consumption. However, on a global basis, the quality of camel meat has received little attention from meat research groups. 

The promotion and preservation of the quality characteristics of camel meat constitute a major challenge for breeders, butchers, and, possibly, manufacturers located in arid regions. In recent years, several studies have been carried out on the use of different methods (biopreservation, modified atmosphere packaging (MAP) and active packaging) to extend the shelf-life of meat and meat products of several species such as camel, beef, and pork [4,5,6]. The search for new alternatives to maintain the quality stability of camel meat has become necessary.

Over the last decades, our group has been actively engaged in laboratory research to replace synthetic additives in food processing with natural ones such as biopreservatives, particularly derived from local plants or from industrial by-products [7,8,9,10]. Bioactive compounds can effectively chelate transition metals, scavenge free radicals, and reduce microbial growth, thus halting progressive oxidative damage and microbial spoilage. The properties of bioactive compounds such as oleuropein, luteolin-7–glucoside, apigenin-7-glucoside, and hydroxytyrosol extracted from *Olea europaea* (leaves, fruits, or olive oil) have been known for several years. Various papers featuring models or animal systems have been published during the last decade [7,11,12,13].

The Laperrine olive tree (*Olea europaea* subsp. *laperrinei* (Batt. and Trab.) Ciferri) (*laper.*OLE) is an endemic and relic *Oleaceae* from the mountainous regions of the central Algerian Sahara comprising the Hoggar mountains and the Tassili n’Ajjer plateau. Its presence for millennia in a vast and isolated environment, where the genetic flows between populations are very limited, ensures that its genetic heritage is particularly well-preserved [14]. The Tassili n’Ajjer is an immense plateau (80,000 km^2^) located at a relatively high altitude (1700 m) compared to the rest of the Sahara. Some in vitro research has already pointed out the importance of this plant in the medical and pharmaceutical fields due to its antiparasitic and anticarcinogenic properties [15,16,17].

Nisin is a normal food constituent due to the presence of many lactic acid bacteria, including *Lactococcus lactis* subsp. *lactis*., in fermented dairy products and meat products, well-documented for its inhibitory effect against various microorganisms [18,19]. Nisin may be effective in controlling microorganisms growth, especially when used in conjunction with other antimicrobial technologies. Nisin could be integrated into processed meats packaging films as antimicrobial to create a bioactive packaging system which will maintain their bioactivity during product long–term storage and thus extend the safety and shelf-life of packaged meats, vacuum-packed sliced cooked ham, minced pork meat, and in ready-to-eat sausage [20,21,22,23].

The results of the assessment support the safety of nisin when used under pre-established conditions. In fact, the use of nisin as a food preservative is authorized in Australia, Canada, New Zealand, Europe and the United States of America (USA). Nisin is also recognized internationally under the Codex General Standard for Food Additives [24].

Consumers often find that the camel meat displayed in retail markets is less tender than meat of other species (beef, goat, and lamb) and exhibits signs of *off-odor* due to microbial and lipid oxidation [25]. The great variability of organoleptic qualities found in camel meat, including wide variations in tenderness and perception, discourage the consumer from frequently buying this type of meat.

In the traditional supermarkets, meat is often sold without packaging in order to readily provide consumers with the product. Trade meat under these conditions would require exceptional hygienic measures to achieve consistent product quality and safety. Therefore, to prevent spoilage caused by the growth of psychrotrophic bacteria, as well as to avoid pigment and lipid oxidation, meat is typically packaged under MAP and treated with bioactive compounds [5,26]. In this context, the packaging of fresh meat under modified atmosphere (MA) with high O_2_ and CO_2_ has raised great interest in terms of improving color and microbial and oxidation stability. This strategy would allow for shelf-life extension while ensuring safety during distribution and subsequent retail display under refrigerated conditions [26]. In a previous study, Jouki and Khazaei [27], when using anoxic MAP (60% CO_2_ + 40% N_2_) alone on camel meat, found that the combined effects of refrigeration (4 °C) storage and MAP improved physicochemical attributes without undesirable effects on its sensory acceptability during 21 days of storage. These results suggest that MAP had a significant impact on the quality of refrigerated camel meat.

Thus, thanks to a possible synergism among hurdles, the application of combined treatments might help to extend the shelf-life of camel meat by reducing the growth of spoilage microorganisms and oxidation reactions, and it might also improve food safety by preventing the growth of pathogens.

Therefore, the aim of the present study was to characterize the Laperrine OLE from the Algerian region of Tassili n’Ajjer, and to investigate the technological potential of a combined application of the leaf extract from that olive tree with nisin in order to promote the quality of higher O_2_ packaged fresh camel meat after long-term storage.

## 2. Materials and Methods

### 2.1. Preparation of Powder and Laperrine Olive Leaf Extract and Nisin

**Figure 1:**
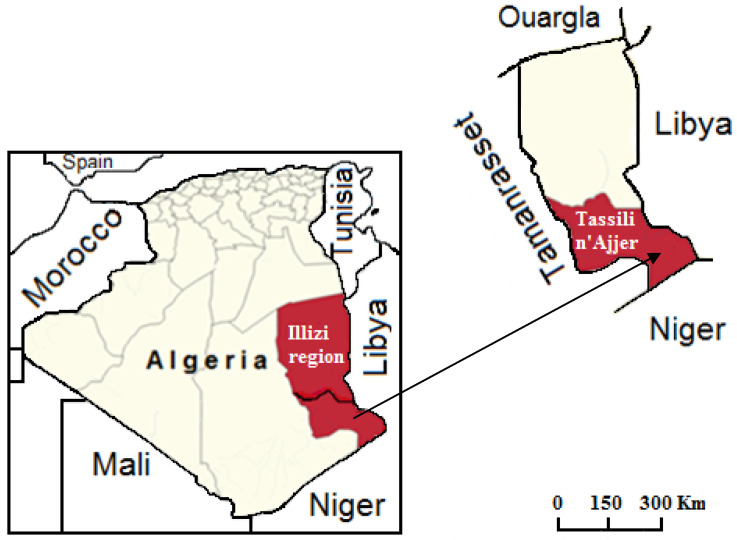
Map showing the Wilaya of Illizi with the geographical location of the study area.

Fresh leaves from wild olive trees (locally called “aléo” in the Tamahaq language: *Olea europaea* subsp. *laperrinei*) were collected in November 2018 in Ihrir (Tassili n’Ajjer, Algeria: Latitude: 25°42 N, Longitude: 8°40 E, Altitude: 1750 m; distance to the Mediterranean Sea: 2065 km) (Figure 1). Leaves were collected from different parts of various trees in the same locality. Harvested leaves were rinsed thoroughly with sterile distilled water, and air dried in the shade at room temperature (~30 °C) for two months. After drying, the dried leaves were immediately vacuum-packed, and stored in the dark at room temperature.

The packaged dried Laperrine olive leaves were ground into powder (size: ~0.1 mm). The extraction was carried out by macerating the olive leaf powder in methanol/water (80:20, *v*/*v*), and the mixture was kept under agitation for 24 h. Insoluble material was removed by centrifugation at 15,000× *g* for 40 min. Subsequently, the clear supernatant was obtained after evaporation of the solvent by a rotary evaporator under vacuum at 40 °C. Then the supernatant was filtered (0.45 μm) to obtain the olive leaf extract (*laper*.OLE), which was stored in light-protected glass vials at −20 °C until further analysis and use.

Nisin 0.0025% (25 ppm) (balanced NaCl) from *Lactococcus lactis* was purchased from Sigma–Aldrich (Madrid, Spain, ref. 5764-1G). Nisin solution was prepared by dissolving nisin (from *Lactococcus lactis*) in sterile distilled water to final concentrations of 25 ppm. Undiluted natural 100% *laper*.OLE was used in this study at 0.05% (500 ppm) and 0.1% (1000 ppm). The choice of 25 ppm of nisin was determined from the scientific literature [24]. However, the two concentrations of *laper*.OLE have been selected through preliminary sensory analysis with different levels of *laper*.OLE to optimize its use on camel steaks.

### 2.2. Estimation of the Total Phenolic Compounds in the Laperrine Olive Leaf Extract

Total phenolic compounds (TPCs) in the *laper*.OLE were determined spectrophotometrically according to the Folin–Ciocalteu method with some modifications [28]. Gallic acid (GA) was used as phenolic compound standard for the calibration curve. Results were expressed as milligrams of gallic acid equivalents per gram of sample dry weight (mg GAE/100 g).

### 2.3. Analysis of Phenolic Constituents in laper.OLE 

Analysis of compounds in the *laper*.OLE extract was performed with an analytical high-performance liquid chromatography unit (Agilent Technologies 1200 series, Santa Clara, CA, USA), equipped with a diode array detector (HPLC/DAD). Quantification of phenolic compounds was done by calibration curves relative to external standards developed by injecting different amounts of a known standard compound in the HPLC column [7].

### 2.4. Camel Meat Treatment and Packaging under MA

The camels used for this trial were cared for in accordance with the guidelines from the Algerian Ministry of Agriculture (Arrêtés 1 August 1984 and 15 July 1996).

Eight healthy female camels (Tergui one-humped camel; *Camelus dromedarius*) were slaughtered at a slaughterhouse at Illizi Province, Algeria. To minimize variability, only animals aged 7–8 years old and weighing 456 ± 15 kg were slaughtered. Whole *Longissimus* muscle (LM) was carefully separated pre-rigor (24 h post-mortem) from carcass. Separated muscles were cooled, wrapped in a plastic film, and transferred directly without delay to the laboratory under ice storage. Upon arrival in the laboratory, meat was washed with sterile deionized water, and depot fat and connective tissues were removed under refrigerated conditions. 

Portions of camel steaks of approximately 100 g (~2 cm thick and ~75 cm^2^ surface) from 8 whole LM were aseptically cut, using sterile cutting boards and knives. In total, 144 steaks were obtained from one slaughter batch and then distributed randomly within the treatment groups. Obtained camel steaks were randomly divided into five groups. The first group consisted of non-treated camel steaks obtained directly from LM; the second group consisted of camel steaks treated with nisin (25 ppm); the third group consisted of camel steaks treated only with a lower amount of *laper*.OLE (500 ppm); the fourth group consisted of camel steaks treated with nisin combined with the lower amount of *laper*.OLE: nisin/*laper*.OLE (25 ppm/500 ppm); and the fifth group consisted in camel steaks treated with nisin combined with a higher amont of *laper*.OLE: nisin/*laper*.OLE (25 ppm/1000 ppm), respectively). Each group contained 24 samples for 6 selected times of sampling and analysis (days 5, 10, 15, 20, 25, and 30 of storage period).

Each group of samples was immersed for 1 min in dipping solution (Nisin, *laper*.OLE, and the different combinations thereof), that was to be added in appropriate volumes to the surface (both sides) of the samples in the related groups. Control samples were immersed in sterile distilled water. The ratio of camel steak samples to chemical solution volume was 3:1 (*v*/*w*). This ratio was selected to allow better immersion of the camel steaks in the solution and; therefore, ensure homogeneous treatment over the entire surface of the samples. The volume of the solution decreases due to the meat absorption, so the initial volume was adjusted each time to keep the same proportion. Draining time of all samples after the treatments was 5 min. Each treated or untreated camel steak was individually packaged into a polystyrene tray. Afterwards, all trays containing samples were completely enveloped with transparent polyethylene/polyamide (PE/PA) coextruded film supplied by the Department of Food Science, Veterinary Faculty, University of Zaragoza (Spain), filled with a gas mixture of 80% O_2_ + 20% CO_2_, and stored at 1 ± 1 °C to mimic storage conditions currently applied in fresh red meat markets in the USA and in the European Union (EU). For each selected sampling time (0, 5, 11, 16, 20, 25 and 30 days), four trays were selected arbitrarily. Two of them were used for MetMb percentage, sensory, and Warner-Bratzler shear force (WBSF) analyses, and the two others were used for microbial analysis and thereafter for thiobarbituric acid-reactive substances (TBA-RSs) analysis. Results were obtained from at least 3 independent experiments carried out on different working days.

### 2.5. pH of Camel Meat

Changes in pH of camel steaks were determined by homogenizing 10 g of the sample with 50 mL of chilled distilled water at 1300 rpm with an Ultra-Turrax T25 homogenizer (Janke and Kunkel, Staufen, Germany). The pH values were measured with a digital pH meter attached with a probe (Crison Instruments, Barcelona, Spain). Three readings were taken by dipping the pH meter probe in the stable homogenate samples and calculating the average. The pH meter was calibrated every six measurements using buffers at pH 4.0 and 7.0.

### 2.6. Lipid Oxidation of Packaged Fresh Camel Meat Treated with laper.OLE and Nisin: Thiobarbituric Acid-Reactive Substances (TBA-RSs)

The thiobarbituric acid method is the one most commonly used for the determination of secondary lipid oxidation compounds in animal products [29]. The method used for our study was developed at the Laboratory of Meat Science and Technology (University of Zaragoza, Spain) as an adaptation of the method of Pfalzgraf et al. [30]. This method has indeed yielded preferable results and has been adopted by corresponding laboratories without any modification. A standard curve was prepared using 1,1,3,3-tetramethoxypropane (MAD). TBA-RSs were expressed as milligrams of malondialdehyde per kilogram of sample (mg MAD equivalents/kg meat).

### 2.7. Pigment Oxidation Analysis (MetMb%) of Packaged Fresh Camel Meat Treated with laper.OLE and Nisin

The percentage of MetMb on the camel steak surface was measured according to Stewart et al. [31], by measuring meat surface reflectance at 525 and 572 nm with a spectrophotometer (Minolta CM-2002; Osaka, Japan). On the initial day of the experiment (day 0), 0% MetMb was estimated by measuring the maximum value of ratios: (K/S) _572 nm_/(K/S) _525 nm_. The value of 100% MetMb was obtained following the same procedure after oxidizing a sample with 1% (*w*/*v*) solution of potassium ferricyanide (C_6_N_6_FeK_3_) during 30 min.

K/S is the ratio of light absorption to light scattering, and is calculated from reflectivity (R_∞_) values using the Kubelka–Munk equation:

K/S = (1 − R_∞_)^2^/2R_∞_ (1)

where R_∞_ is the reflectance factor at complete opacity.

### 2.8. Warner-Bratzler Shear Force of Packaged Fresh Camel Meat Treated with laper.OLE and Nisin

The shear force for the LM samples was assessed using a Warner-Bratzler shear device commonly used in meat technology to estimate the force of any cutting action which splits a product into two fragments as described by Caine et al. [32], with certain modifications. The Warner-Bratzler shear blade measures the force required to cut through the meat simulating the cutting of meat in the mouth during the first bite. This test measures the maximum force (N) required to shear (cut off) a sample of meat as a function of knife movement (mm) and compression. 

Steaks were placed in individual plastic bags and cooked in a water bath (model B21, Fisher Scientific GmbH, Schwerte, Germany) for 60 min at 80 °C, to a final internal temperature of 75 °C. After cooking, samples were overwrapped in polyvinyl chloride (PVC) film and cooled before coring [33]. Prior to texture analysis, samples were kept at room temperature for 3 h. At least six cores of approximately 1 × 1 × 3 cm (height × width × length) from each camel steak were removed parallel to the longitudinal orientation of the muscle fibers. The cores were sheared perpendicularly to muscle fiber orientation using an Alliance RT/5 testing machine (MTS Systems Corp.; Eden Prairie, MN, USA) with a Warner-Bratzler shear device (pre-test speed: 3.0 mm/s; test speed: 2.0 mm/s; post-test speed: 3.0 mm/s). Down stroke distance was 35.0 mm). Shear force data from six rectangular cores were recorded and the average of six readings was used for analysis. Results were expressed as load in kg.

### 2.9. Determination of Total Psychrotrophic and Pseudomonas spp. Counts in Packaged Fresh Camel Meat Treated with laper.OLE and Nisin

Camel steaks were evaluated for surface microbiological quality according to International Commission of Microbiological Specification for Foods (ICMSF) [34] by determining aerobic psychrotrophic microbiota count using plate count agar (PCA, Merck; Darmstadt, Germany) incubated under aerobic conditions (7 °C/10 days). Pseudomonas spp. were enumerated in plates of cephaloridine fucidin cetrimide (CFC) selective agar (Oxoid; Basingstoke, UK) which were incubated at 25 °C/72 h. Background microbiota in the samples immediately after purchasing was also determined. Mean values of counts (log_10_ colony forming units (cfu)/g) from triplicate plates for the same sample were determined.

### 2.10. Sensory Analysis of Packaged Fresh Camel Meat Treated with laper.OLE and Nisin

The biochemical analyses described above were complemented with sensory analysis. Indeed, sensory analysis often proves to be a sensitive method for detecting the presence of residual compounds from the added extract in the matrix, or of derivates of volatile compounds from lipid oxidation and microbial development. Sensory analysis was carried out in individual cabins by 16 semi-trained panelists (5 men and 11 women), aged 20 to 25, who were students in the Food Science program and were familiar with meat consumption. Three open discussion sessions of 60 min were held to familiarize panelists with the “bitterness” attribute and the scale to be used, according to Djenane et al. [7]. In all assessments, the samples were evaluated 30 min after pack opening and maintained in the cold (~2 °C) until evaluation. Steaks were wrapped in aluminum foil and grilled in an electric cooker (internal temperature of 70 °C). Each steak was cut into parts in a prismatic way and in uniform size (10–20 g), and served to panelists in replicates, wrapped in aluminum foil, warm and encoded with three-digit random numbers. For all sessions, samples were presented at room temperature (24 °C) under cool white fluorescent lighting.

For descriptive test, panelists evaluated for each sample, the “bitterness” by using a numerical scale of 1–5 (with 1 = no bitterness and 5 = very bitter) [7]. A score value higher than 3 denoted that camel meat was not acceptable by panelists probably due to high bitterness. Three samples from each group were taken at each selected time for subsequent sensorial analysis. The average of the two replicates was used in the statistical analysis.

### 2.11. Statistical Analysis

All experiments were replicated three times, and the generated data were evaluated statistically by IBM SPSS 21 software (SPSS Inc. Chicago, IL, USA). Analysis of variance (ANOVA) was used to compare the group results. Data are presented as means ± standard deviations. Differences were accepted as significant when *p* < 0.05. Pearson correlations between quality indices of packaged camel steaks treated with nisin and *laper*.OLE and stored at refrigerated temperature were determined.

## 3. Results and Discussion

### 3.1. Total Phenolic Compounds and HPLC-DAD Analyses of laper.OLE

Laperrine olive trees are able to persist under extreme conditions for many years, during which a number of bioactive molecules, including polyphenols, are synthesized. It has been found that the level of phenolic compounds (PCs) accumulated in plants is positively correlated with biotic or abiotic stress, thereby suggesting that these secondary metabolites play a major role in defense mechanisms against such stressful circumstances and microbial attacks. The use of OLE is more prevalently known for its beneficial aspects to health [35,36,37].

Olive leaves have been shown to have a high concentration of PCs [38]. The content of total polyphenols determined by the Folin–Ciocalteu assay for *laper*.OLE was 216.5 ± 2.9 mg GAE /100 g. Hayes et al. [39] found 160.8 ± 2.9 mg GAE/100 g in commercial OLE. According to Mylonaki et al. [40], olive leaves can contain up to 250 mg GAE/100 g of PCs. However, other authors have reported much lower concentrations, for example, 2.8 mg GAE/100 g [41] and 44.3 mg GAE/ 100 g [42].

The TPCs of plant extracts varies in response to different materials, solvents, and extraction methods [38,43,44]. The relation between qualitative and quantitative PCs present in *Olea europaea* leaves been thoroughly investigated. Plant extracts with the highest amount of PCs will be more effective at scavenging free radicals [45]. Wang et al. [17] and Brahmi et al. [46] found that PCs and antioxidant activity of OLE also depend on the variety and the harvest season. Regardless of the leaf dehydration method used, the TPCs of obtained extract can be reduced by 10% [47]. Machado et al. [48] found that dried leaves produce extracts with higher antioxidant capacities than non-dried leaves.

Analysis by HPLC-DAD of *laper*.OLE revealed the presence of seven main compounds (Table 1). Oleuropein is the main compound (63.03%), followed by luteolin-7–glucoside (11.28%), apigenin-7-glucoside (8.15%), and hydroxytyrosol (5.93%).

All these PCs have previously been reported in olive leaves [49]. In another experiment, Djenane et al. [7] quantified various polyphenols found in wild *O. europaea* L. leaf extract, and likewise reported that oleuropein was the main compound (43.25%). Benavente-Garcia et al. [35] also reported that oleuropein was the main compound in *O. europaea* L. leaves present at 24.5%, followed by other PCs. Pereira et al. [50] and Altiok et al. [41] also quantified oleuropein as the most abundant phenolic compound present in a lyophilized and crude OLE, respectively. 

Nonetheless, oleuropein may also act as a plant defense molecule, which is activated by β-glucosidase into the oleuropein aglycone. Polyphenol oxidase and β-glucosidase enzymes were found to be involved in the degradation of endogenous oleuropein in fresh stored olive leaves; oleuropein depletion occurred simultaneously with the formation of the oleuropein aglycon. A linear relationship between oleuropein content and higher antioxidant activity of the extracts from leaves has been previously reported [51]. The effect of the methods used for the freezing and drying of olive leaves on the polyphenol content and biological capacity of the extracts has also been investigated [52].

Apart from such variability factors, the preparation method (dehydration and grinding) also has an effect on PC, along with processes and techniques for qualitative and quantitative analysis thereof [53].

### 3.2. pH of Camel Steaks

Meat pH is an important factor, since it will partially determine the rate of oxidation of myoglobin and lipids, and therefore of the meat itself. Neither packaging with high O_2_/CO_2_ concentrations nor biopreservation treatments significantly (*p* > 0.05) affected the pH of camel meat (pH ranged between 5.7 and 5.9 over the entire period of storage; results not shown).

### 3.3. Lipid Oxidation of Packaged Fresh Camel Meat Treated with laper.OLE and Nisin: Thiobarbituric Acid-Reactive Substances (TBA-RSs)

Camel lipids are distinguished from lipids of other animals (beef, pork, and lamb) by their high content in PUFAs from the omega3 series [2]. These fatty acids play an essential role in human nutrition; they are involved in the prevention of cancer as well as of cardiovascular and inflammatory diseases. However, PUFAs are very sensitive to oxidation reactions, thereby limiting shelf-life during storage due to the development of *off-odor* [54]. *Off-odor* formation in stored meat is known to reduce the product’s sensory quality and thus its acceptance on the part of consumers.

These reactions affect the product’s physico-chemical, organoleptic, and nutritional qualities. The secondary products of lipid oxidation often result from the breakdown of primary products; the most commonly measured secondary products are aldehydes. Thiobarbituric acid reacts with malonaldehyde. However, it also reacts with other compounds that may result from the oxidation of long-chain PUFAs. The term “reactive substances with thiobarbituric acid” (TBA-RSs) is then used.

**Figure 2:**
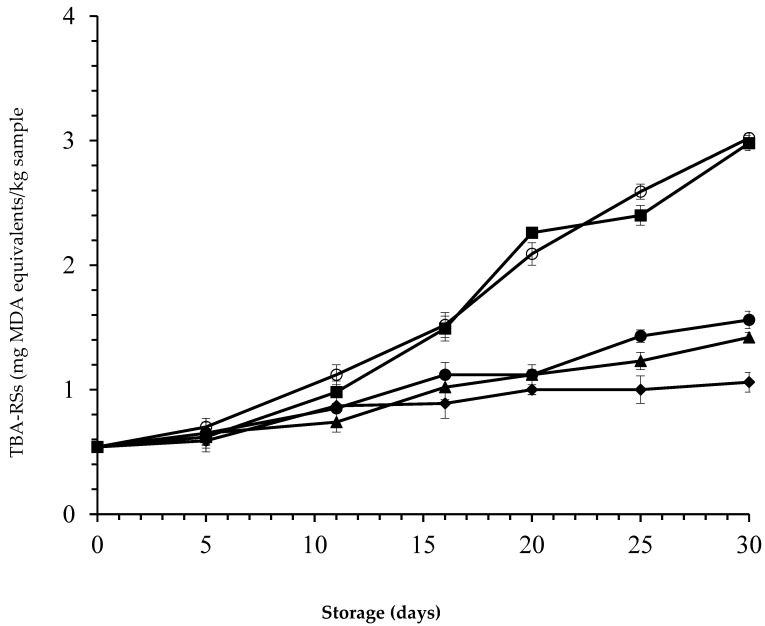
Thiobarbituric acid-reactive substances (TBA-RSs) (mg malonaldehyde/kg meat) in camel steaks stored at 1 ± 1 °C under atmospheres of 80% O_2_ and 20% CO_2_ after treatment with 25 ppm nisin (■); 500 ppm *laper*.OLE (▲); 25 ppm nisin/500 ppm *laper*.OLE (●); 25 ppm nisin/1000 ppm *laper*.OLE (◆); or untreated samples (○). Error bars represent the standard deviation.

Figure 2 shows that the initial TBA-RSs values equal 0.54 mg MAD equivalents/kg for all samples, thereby lying below the limit for animal product standards (1.50–2.00 mg MDA/kg) [7,55].

On day five, all samples contained similar TBA-RSs values (~0.65 mg MAD/kg) (*p* > 0.05); they then gradually increased during the storage period. Indeed, it appears that the levels of TBA-RSs in control and in the samples treated only with nisin showed an abrupt increase in TBA-RSs up to 11 days of storage (*p* < 0.05) and exceeded that limit thereafter, while samples with *laper*.OLE added showed no increase or only a slight increase in TBA-RSs: the threshold was not even reached beyond the end of storage (30 days). A lower degree of formation of TBA-RSs has been achieved in various animal products stored at refrigerated temperature under MAP by treating them with herbal extracts [5,26,53].

In the present study, the packaging of fresh camel meat under MAP coupled with the *laper*.OLE biopreservation method strongly delays the formation of secondary oxidation compounds. However, depending on the type of treatment, the oxidation phenomenon was more or less pronounced. As expected, nisin treatment had no significant antioxidant effect (*p* > 0.05). Analysis of variance showed that the TBA-RSs values in the samples treated with *laper*.OLE and in combination with nisin are significantly different from samples treated only with nisin and untreated samples during the full period of storage (*p* < 0.05). In a similar way, an experiment carried out by Djenane et al. [7] explored the effect of dried powder leaves extracted from the Algerian wild olive tree (1–5%) on the stability of minced beef during retail-display. Moreover, it has been reported that a 5% OLE level has a retarding effect on lipid oxidation in camel meat. A similar decrease in TBA-RSs was observed after 10 days in packed beefsteaks treated with 0.1% rosemary extract [26], and after 14 days of storage of packed beefsteaks with active packaging containing 0.5–4% oregano extract [56]. 

Similar findings were reported by Botsoglou et al. [57] who showed that the addition of OLE delayed lipid oxidation in long-term frozen n-3 fatty acids-enriched pork patties. The antioxidant activity of PCs in OLE could be due to the presence of hydroxyl groups in their structure such as oleuropein, hydroxytyrosol, and luteolin-7-O-glucoside acid as a result of their ability to scavenge oxygen species such as hydroxyl radicals [35]. Free radicals from lipid oxidation can also attack meat proteins; the heme proteins (myoglobin) responsible for the stability of red color in meat are; therefore, affected by this phenomenon due to interactions with lipid oxidation products. An experiment carried out by Taghvaei and Jafari [58] revealed that hydrolysate extracts of olive leaves have a higher protective effect against lipid oxidation than butylated hydroxytoluene (BHT) and butylated hydroxyanisol (BHA). The extract in that study also contained flavonoids (e.g., metabolites), which, according to N’guessan et al. [59], display a significant antioxidant activity. Such antioxidant activity has been explained by two corresponding phenomena: Hydrolysis of oleuropein to hydroxytyrosol with the corresponding increase in the antioxidant capacity of the extract, as well as the synergistic effect of phenols on the whole OLE [35,60,61]. Differences among most herbal extracts in their ability for inhibiting TBA-RSs formation were more likely governed by their differences in composition and structure.

### 3.4. Pigment Oxidation of Packaged Fresh Camel Meat Treated with laper.OLE and Nisin: Metmyoglobin Percentage Analysis 

Changes in color are often the main cause of meat rejection by consumers in retail stores [62]. To avoid this problem, the meat industry has heavily invested in the development of innovative packaging. Traditionally, in Consumer Sales Unit (CSU) systems, the bright color of red meat when wrapped in O_2_ permeable film can only be preserved for a few days (~3 days) at refrigeration temperature. Dromedary meat is described as “raspberry red”, and sometimes as dark in adult animals, due to a higher concentration of myoglobin and high iron content, which can act as a pro-oxidant that causes lipid oxidation [63].

**Figure 3:**
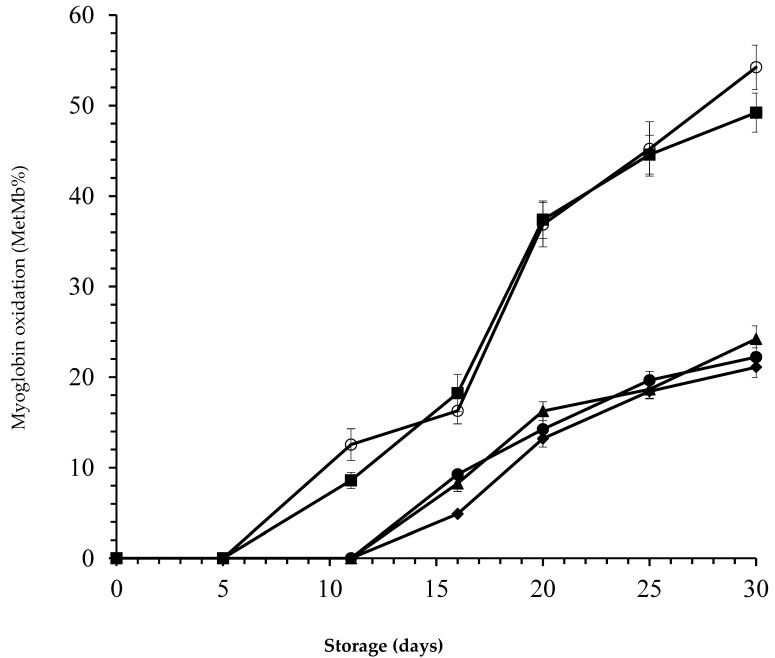
Pigment oxidation (surface metmyoglobin: MetMb%) in camel steaks stored at 1 ± 1 °C under atmospheres of 80% O_2_ and 20% CO_2_ after treatment with 25 ppm nisin (■); 500 ppm *laper*.OLE (▲); 25 ppm nisin/500 ppm *laper*.OLE (●); 25 ppm nisin/1000 ppm *laper*.OLE (◆); or untreated samples (○). Error bars represent the standard deviation.

The content of MetMb (an important pigment associated with color degradation in meat and meat products) was significantly increased for all samples during the storage period (Figure 3).

In *laper*.OLE-treated camel steaks, MetMb was detected only after 11 days of storage (*p* < 0.05). Surface MetMb increased steadily throughout storage for untreated camel steaks (control), and for those treated with nisin alone, reaching a value of 37% at 20 days of storage. However, surface MetMb did not reach 25% in the other samples even at the end of storage (30 days). The presence of nisin had no additional effect (*p* > 0.05). Most important is the fact that a MetMb% value of 40% was obtained on the twentieth day of storage for control samples and those treated with nisin; this value has been demonstrated to be the limit between red and brown color perception by consumers [62]. Maqsood et al. [63] reported that redness values (CIE a*) were higher in vacuum-packed camel meat (22.0) when compared to air-packed (13.73) and wrapped samples (14.7).

The packaged samples treated with *laper*.OLE displayed a greater degree of color stability over the entire period of storage compared with the other samples. Such a protective effect of OLE against color deterioration has also been reported by Hayes et al. [11] in bovine muscle during refrigerated storage. The most likely reason is the high amount of polyphenols present in OLE. 

Djenane et al. [5,26] reported that long term-storage induces the oxidation of pigment (oxymyoglobin: MbO_2_ = bright red color) into brown MetMb. This change decreases the meat’s CIE a* values and makes it unacceptable for consumers. Lipid oxidation has also been proposed as a factor responsible for decrease in meat redness, especially during prolonged exposure to air. The improved CIE a* stability of red meats packaged under conventional MA has also been reported by authors applying different biopreservation methods: This effect could be attributed to the presence of bioactive molecules in plant-based extracts, thereby inhibiting myoglobin oxidation and, subsequently, the formation of MetMb on the meat surface.

According to Mancini and Hunt [64], consumer rejection of altered discolored meats can be the source of significant economic losses estimated at several million dollars ($)/year. In an ambient atmosphere (21% O_2_) or in a superoxygenated atmosphere (60–80% O_2_), the red color of meat is due to the oxygenation of the pigment Mb into oxymyoglobin (MbO_2_). This oxygenation is reversible as a function of the partial pressure of O_2_ (*pp*O_2_) exerted on the surface of the product. The discoloration or even browning of the surface of meat results from the oxidation of that pigment to brown-colored metmyoglobin (MetMb). Many studies have reported the beneficial effects of conventional and non-conventional MA for the packaging of meat and meat products [65,66,67,68].

### 3.5. Warner-Bratzler Shear Force of Packaged Fresh Camel Meat Treated with laper.OLE and Nisin

Tenderness of red meat is a very important issue for the meat industry; consumers expect the meat they purchase to be homogeneously tender over time. Toughness is attributed to various factors including the amount of intramuscular connective tissue, intramuscular fat, and the post-mortem ageing period [69,70]. For the evaluation of meat tenderness, many mechanical tests are available. Camel meat is commonly considered as tough compared to other meats because it is mainly obtained from older animals. 

The effect of *laper*.OLE treatment on the instrumental texture of packaged camel steaks is shown in Table 2. Neither MAP nor biopreservation methods significantly modified the tenderness of camel steaks as compared to control samples (*p* > 0.05), expressed in term of WBSF. However, storage time did have a significant effect on WBSF (*p* < 0.05). On the initial day of storage, a similar shear force was observed for all camel steaks. However, by day 20 of storage, all steaks were only moderately tender, and WBSF was reduced up to 21.60% by day 30 of storage. 

The proteolysis that takes place during long term storage is probably the major factor that contributed to the variation in shear force tenderness observed among different camel steaks. A previous experiment by the same research group evaluated the impact of active packaging with oregano extract on the textural profile of MAP beef [5]. In that experiment, WBSF was particularly reduced in the course of long-term storage. Camel meat is probably one of the meats whose tenderness is not yet one of the primary decision criteria for consumers, for several reasons: On the one hand, slaughter is almost always practiced on older animals; on the other hand, traditionally there is only a weak tendency to consume camel meat “as is”.

The need to define a consumer threshold for meat acceptability remains vital. Several studies have been carried out using a trained panel to establish threshold values of WBSF for tenderness acceptability [71,72]. These thresholds allow the tested muscles to be placed in different classes of tenderness. On the other hand, many authors have attempted to determine the combination of several tenderness indicators (collagen level, types of fiber, enzyme concentrations, sarcomere length, ageing duration, etc.) that would make possible to predict tenderness. In addition, traditional methods for the analysis of muscle characteristics are time-consuming and expensive; they cannot be automated and are not efficient enough to meet the constraints of industrial use. Among novel methods explored for the measurement of tenderness indicators, genomics currently holds a large place. Zahedi et al. [73] reported a higher correlation of biomarkers with physicochemical and quality properties of camel meat. 

### 3.6. Microbiological Counts in Packaged Fresh Camel Meat Treated with laper.OLE and Nisin

Fresh meat is highly susceptible to microbial spoilage. The main factor limiting its microbial shelf-life during subsequent aerobic storage is the activity of microorganisms. This incidence is of special concern in sale meats in the Algerian Sahara due to probable temperature abuse conditions. The nature of microbial association and their loads depend on the preliminary meat contamination and on the specific storage conditions that can affect the development of the type and rate of the spoilage bacteria [74]. *Pseudomonas* spp. and total psychrotrophic microbiota (TPM) are a major index for microbiological shelf-life estimation of animal products during processing and storage. Lactic acid bacteria are widely represented within the group of psychrotrophs. Particularly, *Lactobacillus* spp., *Carnobacterium* spp., and *Leuconostoc* spp. are associated to the spoilage of refrigerated raw meat [75]. Among the other psychrotrophic bacteria, the species *Brochothrix thermosphacta* and *Enterobacteriaceae*, that mainly belong to the genera Enterobacter, Serratia, and Hafnia, are an important meat spoilage bacterium and commonly associated with the spoilage of fresh meats [76,77]. Generally, the normal spoilage microbiota of the meat was initially present in low counts and with regard to best practices, the starting total microbiota could be approximately 3 log_10_ cfu/g [66,78]. Nevertheless, this value is only indicative and refers here to the total viable microbiota. Meat spoilage needs to be assessed to the genus-species level, because potentially protective bacteria can also occur in meats.

**Figure 4:**
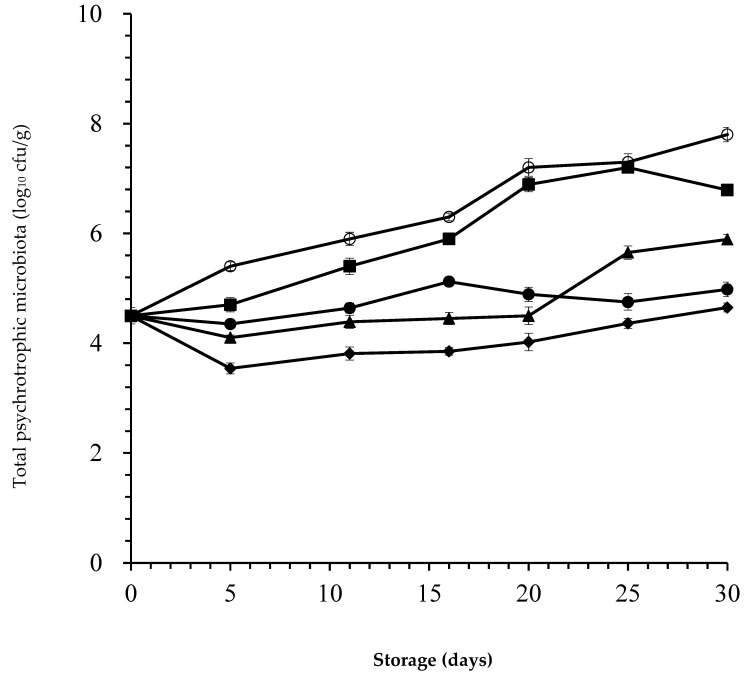
Numbers (log_10_ cfu/g) of total psychrotrophic microbiota (TPM) recovered from camel steaks stored at 1 ± 1 °C under atmospheres of 80% O_2_ and 20% CO_2_ after treatment with 25 ppm nisin (■); 500 ppm *laper*.OLE (▲); 25 ppm nisin/500 ppm *laper*.OLE (●); 25 ppm nisin/1000 ppm *laper*.OLE (◆); or untreated samples (○). Error bars represent the standard deviation.

In our study, the initial TPM load of 4.50 log_10_ cfu/g obtained in camel meat was far from the normal microbial count for fresh meat (Figure 4). The microbial results obtained herein revealed the possible lack of proper hygienic measures adopted during the slaughtering and processing of the studied camel meat, leading to poor initial microbial quality of the product. The limited shelf-life of fresh meat is due to the initial levels of spoilage microbial contamination transferred to the surface muscle during slaughter, dressing and boning. 

After two weeks of storage, the number of TPM in untreated camel steaks was around 7 log_10_ cfu/g. However, all camel steaks treated with *laper*.OLE were below 6 log_10_ cfu/g even at the end of storage. The lower-dose treatment with *laper*.OLE (500 ppm) significantly reduced TPM growth by 1.65 and 1.91 log_10_ cfu/g on days 25 and 30 of storage, respectively. Such levels reductions were increased to 2.55 and 2.82 log_10_ cfu/g, respectively, after combination of lower-dose *laper*.OLE with nisin. Furthermore, the combination of higher-dose *laper*.OLE (1000 ppm) with nisin significantly reduced (*p* < 0.05) TPM in comparison to control samples by 3.20, 2.95, and 3.15 log_10_ cfu/g on days 20, 25, and 30 of storage, respectively.

Figure 4 shows that lower-dose treatment with *laper*.OLE (500 ppm) alone leads to a lower bacterial cell count than with *laper*.OLE (500 ppm) + nisin up to day 20. Thus, these results question the synergistic effect of the lower-dose of *laper*.OLE and nisin treatment on the TPM. As a result, the lower-dose of *laper*.OLE itself might be sufficient to prevent the spoilage and extend the shelf-life of camel steaks.

However, in the presence of a higher concentration of OLE, the effect of this combination was evident throughout the storage and; therefore, there is no doubt about its antimicrobial effect. Nisin exerted a complementary antimicrobial activity with regard to the TPM, thereby demonstrating this formulation’s potential use to improve the microbial quality of packaged product (*p* < 0.05). Gharsallaoui et al. [19] already found that nisin has strong antimicrobial effects on meat and meat products when used alone or in combination with other antimicrobials. The same findings were pointed out by Tang et al. [6] who found that nisin combined with gingerol significantly reduced microbial growth and subsequent formation of biogenic amines in the meat and edible offal of camel. The highest dose of *laper*.OLE (1000 ppm) combined with nisin (25 ppm) kept TPM counts below 5 log_10_ cfu/g values, even at the end of storage (30 days). Similarly, Djenane et al. [7] reported a net reduction of the TPM in treated minced beef with OLE during display depending on the concentration used. Meat is often considered microbiologically spoiled when a total microbial count of 7 log_10_ cfu/g is exceeded. 

**Figure 5:**
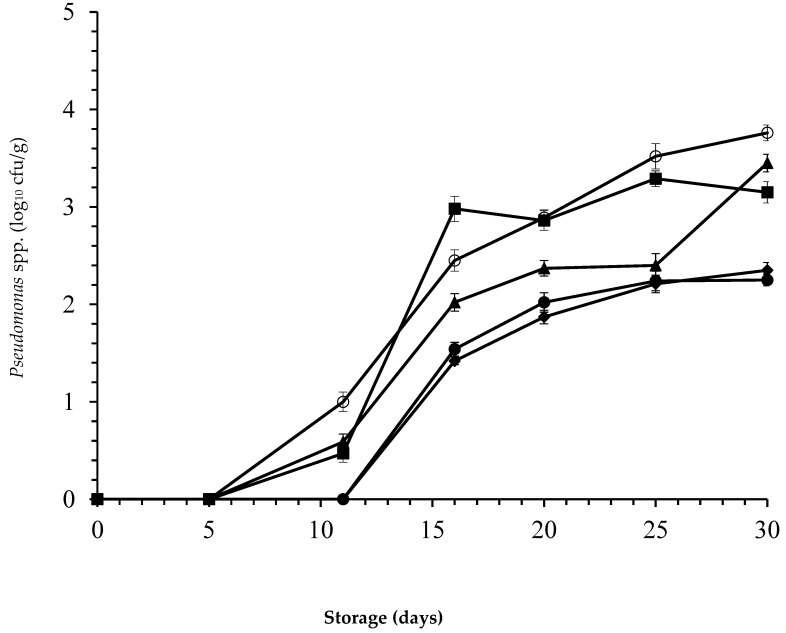
Numbers (log_10_ cfu/g) of *Pseudomonas* spp. recovered from camel steaks stored at 1 ± 1 °C under atmospheres of 80% O_2_ and 20% CO_2_ after treatment with 25 ppm nisin (■); 500 ppm *laper*.OLE (▲); 25 ppm nisin/500 ppm *laper*.OLE (●); 25 ppm nisin/1000 ppm *laper*.OLE (◆); or untreated samples (○). Error bars represent the standard deviation.

A *Pseudomonas* spp. population was detectable only after the 11th day of storage in both combined treatments. After two weeks of storage, untreated samples showed higher counts than treated ones (*p* < 0.05). In control samples after 20 days of storage, the population count of *Pseudomonas* spp. reached 3.5 log_10_ cfu/g (Figure 5). The treatment with nisin alone exerted a moderate antibacterial effect. However, *Pseudomonas* spp. counts in samples treated with *laper*.OLE combined with nisin remained below 2.5 log_10_ cfu/g during the entire storage period.

A clear influence of combined treatments on *Pseudomonas* spp. population can be observed. At the highest dose of *laper*.OLE combined with nisin, the reductions of *Pseudomonas* spp. population compared with untreated samples were 1.02, 1.31, and 1.41 log_10_ cfu/g at 20, 25, and 30 days of storage, respectively. *Pseudomonas* spp. counts are very low and most likely not responsible for any changes in the sensory attributes of packaged camel meat. This maximum protective behavior was also probably favored by the presence of 20% CO_2_ incorporated in the atmosphere packaging. 

The characterization of the isolates from total psychrophilic spoilage microbiota affected by storage conditions not only at the species level but also at the strain rank is also an important matter that has been increasingly considered by microbiologists. To understand meat spoilage from different strains of the same species, this approach could potentially play a pivotal role. During the last decades, the crucial comprehension of the microbial association during meat storage has been acquired by using traditional methods. In recent years, the development and application of potent molecular techniques have contributed to produce reliable data on the microbial species and strains occurring during meat spoilage [79,80].

The biological activities of bioactive compounds contained in OLE have been known for several years in model or food systems (turkey breast fillets, flour) [11,50,81]. Djenane et al. [7] recently found that wild OLE from Algeria displayed a high antibacterial activity, probably due to its high content of oleuropein and other compounds detected by HPLC-DAD. Bisignano et al. [82] had previously described hydroxytyrosol as an antimicrobial agent against a broad range of bacteria: It showed high antimicrobial activity against Gram-negative and -positive bacteria more effectively than oleuropein. Hayes et al. [11] studied the antimicrobial activity of OLE in bovine and porcine muscle model systems and demonstrated its antimicrobial effects. The microbiological effects of OLE could be attributed to synergistic phenomena among olive bioactive phenols. Several studies have revealed a higher antimicrobial potential for the oleuropein aglycone compared to the oleuropein glycoside: aglycone inhibited several Gram-negative and -positive bacteria. It is possible that, during industrial processing and heat treatments of olive tree derivates (leaves, fruits or olive oil), the enzymes responsible for the hydrolysis of oleuropein-glycoside to oleuropein-aglycone might be inactivated. Synergistic or even antagonistic effects on other more active antimicrobial compounds from *O. europaea* remain to be elucidated. This is especially important regarding the possible development of a natural extract from *O. europaea* for food preservation. The evaluation of nisin as an antimicrobial has been carried out in several food matrices; it displayed a variable antimicrobial activity in food [83,84]. Our findings present a significant advantage in terms of microbiological stability and subsequently extended shelf-life of camel steaks. 

The mechanism of the inhibitory effect of nisin is mainly due to the prevention of cell wall synthesis [85]. The antimicrobial mechanism of OLE could be explained by the action of biophenols in the disintegration of the bacterial envelopes, leading to ion leakage and ATP depletion. In addition, bioactive compounds of *laper*.OLE, especially oleuropein and hydroxytyrosol, might also chelate some metal ions required for microbial growth. Therefore, *laper*.OLE not only showed antioxidative activity but also displayed antimicrobial properties against spoilage bacteria in camel meat.

### 3.7. Bitterness of Packaged Fresh Camel Meat Treated with laper.OLE and Nisin

Sensory evaluation is often regarded as a very useful tool for the qualitative evaluation of foods. In general, an increase in the level of chemical, microbiological, or physical alteration of the food matrix results in changes in sensory attributes. This corroborates well with the lower values for TBA-RSs, MetMb%, and microbial growth observed in our study.

Since the antioxidant and antimicrobial effects of *laper*.OLE are promising, it would be essential to investigate the sensory impact of residual extract in future food application studies. Among *laper*.OLE compounds, oleuropein is known to express higher bitterness, whereas hydroxytyrosol is known to be non-bitter [86].

The camel steak samples from the different groups were presented simultaneously to panelists for evaluation according to bitterness intensity due to the presence of *laper*.OLE. A score of 1 corresponds to “no bitterness” intensity perceived by the jury and a score of 5 corresponds to “very bitter” (Table 3).

*Off*-*odors* and MetMb accumulation on surface meat frequently reflect the oxidative status of the product [5]. Oxidation of lipids then leads to the formation of aldehydes (TBA-RSs) involved in the degradation of odor and flavor, in particular via the appearance of rancid “*off*-*odor*” or, in the cooked state, “*off*-*flavor*”. On the other hand, the oxidation of myoglobin results in an accumulation of brown pigments on the surface of the product (MetMb).

As expected, the treatment with *laper*.OLE reveals low initial bitterness defects which decrease in intensity over storage time (Table 3). Spoiled samples were not subjected to sensory evaluation. This is particularly the case for control and nisin-treated samples kept after 20 days, because of the odor due to oxidative rancidity and microbial development.

The results obtained herein indicate a good correlation between chemical (TBA-RSs), microbiological (TPM, *Pseudomonas* spp.), and instrumental (color) measurements. In a similar manner, Djenane et al. [7] explored the effect of OLE on the sensorial stability of minced beef during storage. The authors highlighted the association of sensory attributes with purchase intention, concluding that minced beef treated with 5% OLE resulted in higher scores in terms of bitterness, *offf*-*odor*, and overall acceptability than untreated samples. Similar results were obtained by Abdel-Naeem and Mohamed [4] who used ginger extract in minced camel.

### 3.8. Correlations and Shelf-Life Status of Packaged Fresh Camel Meat Treated with laper.OLE and Nisin

Cold storage slows down undesirable alteration factors in animal products, but it might not sufficiently extend the shelf-life of the product throughout the commercial chain. Microbial spoilage, as well as color change coupled with lipid and pigment oxidations, are the critical factor limiting the shelf-life and consumer acceptability of the animal products displayed in refrigerated conditions. 

Correlation coefficients were determined to estimate the degree to which overall acceptability scores are related to other quality attributes (data not shown). The overall acceptability of packaged camel steaks was most highly related with TBA-RSs value, MetMb%, and microbial load (*r* ≥ 0.87). Individual attributes were strongly correlated with one another (*r* ≥ 0.69), demonstrating that an individual improvement of these attributes could bear an influence on other attributes and perceptions. All these factors have an influence on the shelf-life of packaged meat. Therefore, natural antioxidants, especially phenolic bioactive compounds from laper.OLE, could retard lipid oxidation and microbial growth; they were also effective in maintaining the sensorial quality of packaged camel meat during refrigerated storage. The rapid expansion of the global market for herbal medicines has led to concerns over the safety and quality of these products. According to the World Health Organization (WHO) [87], plant materials are particularly prone to microbial contamination, and represent a direct health risk to consumers [88,89], since contaminated materials can also lead to the spoilage of food items to which they are added. The improvement of the microbial quality of olive aerial parts, without affecting the composition of their bioactive molecules, should; thus, also be taken in consideration. Our collected fresh olive leaves were dried in the shade for two months. During the process of drying, excessive water was evaporated destroying microbial activity to prevent the alteration and safety purpose. After drying, the dried leaves were immediately vacuum-packed and stored in the dark at room temperature. Indeed, dried spices can be subjected to contamination by bacteria and especially by yeasts when the good manufacturing and storage practices are not respected. Spices and herbs are present in most ready-to-eat products and are often used by the consumer for flavoring purposes without further processing. In our work, we did not perform microbiological laboratory analyzes of our leaf samples. However, the best practices were adopted to avoid any contamination. Moreover, packaging materials and trays might also be a cause of contamination because they were not sterile. Further studies on microbial contamination of olive leaves, materials packaging, and trays are required. 

## 4. Conclusions

Camel meat is widely consumed in many forms because of its attractive price and its popularity among the local Tuareg population. However, under poor conditions of sale and storage the product’s shelf-life can be drastically reduced. Treatment with *laper*.OLE alone, or preferably combined with nisin, can be a promising tool and constitute a relevant strategy to control microbial growth and oxidation phenomena during the storage of packaged camel meat. This kind of treatment could work in favor of distribution and retail sales under extreme conditions, like those found in Tassili n’Ajjer. In addition, *laper*.OLE alone, or combined with nisin, did not cause a noticeable textural (shear force) effect in camel meat during refrigerated storage. Moreover, nutritional advantages can also be obtained from these new food products by improving bioactive molecule content. 

To the best of our knowledge, this is the first study focusing on antimicrobial and antioxidant activities in camel meat of combined MAP, *laper*.OLE extracts, and nisin. Further studies in other food matrices are required to identify or confirm the synergism between the tested active compounds in order to produce optimized shelf-life and safety effects in conjunction with high organoleptic attributes of food products. 

## Figures and Tables

**Table 1:** Chemical composition (mg/g) of *laper*.OLE.

Compounds	mg/g	%
Hydroxytyrosol	1.63	5.93
Tyrosol	0.07	0.25
Catechin	0.10	0.36
Caffeic acid	0.20	0.72
Rutin	0.37	1.35
Luteolin-7-glucoside	3.11	11.28
Verbascoside	0.79	2.85
Apigenin-7-glucoside	2.24	8.15
Diosmetin-7-glucoside	0.21	0.77
Oleuropein	17.36	63.03
Luteolin-4-glucoside	1.46	5.30
**Total**	**27.54**	

**Table 2:** Warner-Braztler shear force (WBSF) (value = mean ± SD) on camel steaks treated with combined *laper*.OLE and nisin during storage under modified atmosphere packaging (MAP).

Higher O_2_/CO_2_ Packaging Storage Period							
Treatments	0	5	11	16	20	25	30
Control	8.75 ± 0.11 ^aW^	8.60 ± 0.13 ^aW^	8.50 ± 0.12 ^aW^	8.09 ± 0.18 ^abW^	7.40 ± 0.16 ^bW^	6.85 ± 0.11 ^bW^	5.85 ± 0.17 ^cW^
Nisin ^∗^	8.75 ± 0.16 ^aW^	8.73 ± 0.10 ^aW^	8.45 ± 0.14 ^aW^	8.30 ± 0.17 ^aW^	7.13 ± 0.16 ^abW^	6.50 ± 0.12 ^bW^	5.79 ± 0.12 ^cW^
*laper*.OLE ^∗∗^	8.75 ± 0.14 ^aW^	8.65 ± 0.14 ^aW^	8.50 ± 0.17 ^aW^	8.45 ± 0.10 ^aW^	7.53 ± 0.11 ^abW^	6.41 ± 0.11 ^bW^	5.89 ± 0.12 ^bcW^
Nisin/*laper*.OLE	8.75 ± 0.12 ^aW^	8.76 ± 0.14 ^aW^	8.35 ± 0.16 ^aW^	8.41 ± 0.15 ^aW^	7.04 ± 0.12 ^bW^	6.72 ± 0.15 ^bW^	5.68 ± 0.16 ^cW^
Nisin/2 × *laper*.OLE	8.75 ± 0.14 ^aW^	8.64 ± 0.13 ^aW^	8.55 ± 0.14 ^aW^	8.45 ± 0.14 ^aW^	7.80 ± 0.13 ^abW^	6.46 ± 0.10 ^cW^	5.65 ± 0.14 ^cW^

^∗^ Nisin = 25 ppm. ^∗∗^ *laper*.OLE = 500 ppm ^(a–c)^ Means of the same row (between days of storage) with different letters differ significantly (*p* < 0.05). ^(W)^Means of the same column (between treatments) with the same letter no differ significantly (*p* > 0.05).

**Table 3:** Sensory scores (bitterness) of treated and untreated camel steaks packed under modified atmosphere (MA) during period of refrigerated storage.

Storage Period							
Treatments	0	5	11	16	20	25	30
**Bitterness**							
Control	1 ^a^	1 ^a^	1 ^a^	1	1	nd	nd
Nisin ^∗^	1 ^a^	1 ^a^	1 ^a^	1	1	nd	nd
*laper*.OLE ^∗∗^	1.25 ± 0.45 ^ab^	1.19 ± 0.40 ^a^	1.13 ± 0.34 ^a^	1	1	1	1
Nisin/*laper*.OLE	1.31 ± 0.48 ^ab^	1.25 ± 0.45 ^ab^	1.19 ± 0.40 ^a^	1	1	1	1
Nisin/2 × *laper*.OLE	2.31 ± 0.40 ^c^	1.69 ± 0.48 ^b^	1.50 ± 0.52 ^b^	1	1	1	1

Results for the sensory scores (value = mean ± SD) of 16 observations. ^∗^ Nisin = 25 ppm. ^∗∗^ *laper*.OLE = 500 ppm. nd = not determined: *Off*-*odor* and *off*-*flavor* due to oxidative rancidity and microbial development are the causes. ^(a–c)^Values in the same column (between treatments) not having the same superscript letter are significantly different from one another (*p* < 0.05). A score value < 3 of bitterness attribute denoted that camel steaks were deemed acceptable by panelists, thus corresponding with improved shelf-life.
